# Protective Effects of Crocin on Ischemia-reperfusion Induced Oxidative Stress in Comparison With Vitamin E in Isolated Rat Hearts

**DOI:** 10.17795/jjnpp-17187

**Published:** 2014-04-06

**Authors:** Mahin Dianat, Mahdi Esmaeilizadeh, Mohammad Badavi, Ali Reza Samarbaf-zadeh, Bahareh Naghizadeh

**Affiliations:** 1 Department of Physiology, Physiology Research Center, Faculty of Medicine, Ahvaz Jundishapur University of Medical Sciences, Ahvaz, IR Iran; 2 Department of Physiology, Diabetic Research Center, Ahvaz Jundishapur University of Medical Sciences, Ahvaz, IR Iran; 3 Department of Virology, Faculty of Medicine, Ahvaz Jundishapur University of Medical Sciences, Ahvaz, IR Iran; 4 Department of Pharmacology, Faculty of Medicine, Ahvaz Jundishapur University of Medical Sciences, Ahvaz, IR Iran

**Keywords:** Crocin, Vitamin E, Rats, Antioxidants, Ischemia-Reperfusion Injury

## Abstract

**Background:**

Myocardial Injury caused by ischemia-reperfusion leads to cardiac dysfunction, tissue injury and metabolic changes. The production of reactive oxygen species (ROS) and lipid peroxidation are accompanied by ischemia-reperfusion injury.

**Objectives:**

The aim of this study was to assess the cardio protective potential effects of crocin in comparison with vitamin E on antioxidant capacity in ischemia-reperfusion of isolated rat hearts.

**Materials and Methods:**

Seventy male Sprague-Dawley rats were randomly divided into seven groups, including: sham, control and experimental groups treated with different doses of crocin (10, 20 and 40 mg/kg) or vitamin E (100 mg/kg) and a combination of crocin (40 mg/kg) with vitamin E (100 mg/kg) that were administrated orally for 21 days. The heart was quickly excised, transferred to a Langendorff apparatus at constant pressure and subjected to 30 minutes of global ischemia followed by 60 minutes of reperfusion. Cardiac damage markers and antioxidant enzymes were measured.

**Results:**

The results showed that superoxide dismutase and catalase enzyme activities increased and Mallon de aldehyde (MDA) decreased in animals pretreated by crocin (40 mg/kg) and vitamin E (100 mg/kg). Moreover, there was a significant improvement in post ischemic recovery of antioxidant capacity during reperfusion in rats receiving a combination of crocin (40 mg/kg) and vitamin E (100 mg/kg).

**Conclusions:**

The results demonstrated the protective role of crocin on antioxidant capacity, which may partially be related to stability or amplification of antioxidant systems. Like vitamin E, crocin may be beneficial for prevention or treatment of cardiac dysfunction and myocardial infarction in patients with ischemic heart disease.

## 1. Background

Ischemia-reperfusion injury occurs by the production of free radicals in tissues such as the heart, lungs and brain (1). Ischemic heart disease associated with high mortality, and reperfusion therapy has an important therapeutic plan for patients with acute myocardial infarction, but depression in contractile function and change in gene expression and metabolism, frequently occur after coronary reperfusion and lead to harmful complications (2). Therefore, it is important to develop novel therapeutic strategies for ischemia-reperfusion injury. Global ischemia followed by reperfusion in isolated rat hearts was associated with changes in hemodynamic, electrophysiology and antioxidant capacity (3). Cellular ATP reduction causes pathophysiological events, leading to a series of morphologic, biochemical and physiologic derangements. Reactive oxygen species (ROS), which increase after reperfusion, are one of the most important factors in induction of cardiac ischemic-reperfusion injury that cause oxidative damage to cellular macromolecules including membrane lipids, proteins and nucleic acids (4). Recently, the protective action of antioxidants on myocardial infarction in rats has been shown (5). Antioxidants not only decrease the formation of ROS but also have a mediatory effect on the survival and death signaling of ROS (6). Scientists have tried to focus on newer antioxidants to prevent ischemia-reperfusion injury and keep cell homeostasis. Catalase, superoxide dismutase, and glutathione peroxidase as endogenous enzymes and α-tocopherol and vitamin C (as no enzymatic factors) are cellular defenses against (ROS) injury (7). Therapeutic effects of many plants and their extracts in decreasing the incidence of cardiovascular disease and arthrosclerosis have been reported (8). *Crocus sativus* L. known as saffron, belongs to the Iridaceae family and has a dried red stigma, which is used in folk medicine for its various purposes including anti-spasmodic, eupeptic, gingival, sedative, anti-catarrhal, carminative, diaphoteric, expectorant, stimulant, stomachic, aphrodisiac and emmenagogue effects (9). Saffron contains many compounds considered pharmacologically active such as crocin, picrocrocin and safranal. Crocin, a water soluble carotenoid has antitumor (10), radical scavenging (11), anti-hyper lipidemic (12) and memory improving effects (13). Moreover, crocin has antioxidant effects in ischemia-reperfusion models of stroke in rat brains (14) and renal injury (15). Vitamin E is an antioxidant, which is present in the plasma and the cell membrane. Some studies have indicated that vitamin E reduces smooth muscle cell proliferation (16), platelet aggregation (17) and protein kinase C activation (18).

These results indicate that the beneficial effects of vitamin E in acute ischemic syndromes occur through the reduction of oxidative stress (3). Antioxidants work effectively when they are used in combination with each other. With a suitable combination, they can perform a wide range of metabolic activities, free radical scavenging and preventative actions. Several studies have indicated that a combination of vitamins with other antioxidants produces synergistic effects (19). Evidence indicates that crocin has a synergistic antioxidant effect antioxidant effect with α-tocopherol in decreasing of human low lipoproteins (20).

## 2. Objectives

The present study was performed to examine the effect of crocin and compare its potency with vitamin E on antioxidant capacity in isolated rat hearts.

## 3. Materials and Methods

### 3.1. Chemicals

Crocin was purchased from Fluka (Japan), vitamin E was obtained from Sigma-Aldrich Co. (St. Louis, MO, USA). Ketamine (10%) and xylazine (2%) were purchased from Alfasan Co. (Holland). GPX, MDA, SOD kits were purchased from the Randox Company (England).

### 3.2. Animals

Male Sprague-Dawley rats weighing between 200 and 250 g were obtained and approved by the Laboratory Animal Unit of Ahvaz Jundishapur University of Medical Sciences, Ahvaz, IR Iran. They were kept in an animal house with light: dark cycle of 12 hours. The animals were fed chow pellets and allowed water ad libitum. The investigation was approved by the Animal Ethics Committee of Ahvaz Jundishapur University of Medical Sciences (No. ajums.REC.1392.90, Date: May 04, 2013).

### 3.3. Experimental Groups

The animals were randomly divided in to seven experimental groups, with ten rats in each group; including, sham (no global ischemia) control group (underwent global ischemia) and experimental groups treated with crocin (10, 20 and 40 mg/kg), vitamin E (100 mg/kg) and combination of crocin (40 mg/kg) with vitamin E (100 mg/kg). In the sham and control groups, rats were administered saline orally (3 mL/kg/day) using an intragastric tube for 21 days. In the treatment groups, rats were administered crocin orally once a day for three weeks (21) and vitamin E orally for a period of 30 days (8). Normal saline was used as a vehicle.

### 3.4. Induction of Ischemia Reperfusion

Rats were anesthetized by Ketamine HCl (50 mg/kg) and andxylazine (5 mg/kg) containing heparin (1000 U/kg, I.P). The trachea was cannulated and the animals were ventilated using a rodent ventilator (UGO BASILE, model: 7025). The chest was opened and a steel cannula was placed into the aorta and fixed with a suture. Then, the heart was quickly excised and transferred to a Langendorff apparatus while continuously perfused retrograde with Krebs-Henseleit buffer (NaCl 115 mM, KCl 4.6 mM, KH_2_PO_4_ 1.2 mM, MgSO_4_ 1.2 mM, CaCl_2_ 2.5 mM, NaHCO_3_ 25 mM, and glucose 11 mM) equilibrated with 95% O_2_ + 5% CO_2_, pH of 7.3-7.4 at a constant pressure of 70 mmHg and a temperature of 37˚C. The cardiac function was monitored continuously by a power lab system (AD Instruments, Castle Hill, Australia) (22). All hearts were perfused for 20-25 minutes before the induction of ischemia to allow stabilization. All hearts were subjected to 30 minutes of global ischemia, followed by 60 minutes of reperfusion. The successful induction of ischemia was determined by elevation of the S-T segment on the electrocardiogram.

### 3.5. Biochemical Studies

#### 3.5.1. Measurement of Cardiac Marker Enzymes

Coronary effluent was collected at 5, 15 and 60 minutes after reperfusion in order to measure lactate dehydrogenase (LDH), creatinkinase (CK) and creatin kinase-myocardial (CK-MB). Enzyme activity was determined spectrophotometrically, using assay kits from sigma-aldrich (USA) and data was expressed as units per liter (23).

#### 3.5.2. Thiobarbituric acid reactive species

TBARS (thiobarbituric acid reactive species) levels in the heart homogenate were measured as a marker of lipid peroxidation using the method of Ohkawa et al. (24). The absorbance was read at 532 nm. Unit was expressed as nmole per mg protein (24).

#### 3.5.3. Endogenous Antioxidant Enzymes in Heart Homogenate

The heart was removed and frozen in liquid nitrogen and stored at -80°C for measuring superoxide dismutase (SOD) and catalase (CAT) enzymes. Frozen tissue samples were quickly homogenized in 50 Mm ice-cold 10% phosphate-buffers saline and centrifuged at 14000 × g for 15 minutes at 4°C. The supernatants were separated and used for enzyme activities assays (4). Superoxide dismutase and catalase in the homogenate were estimated according to Marklund (25), Aebi (26) and Rotruk et al. (27).

#### 3.5.4. Ferric Reducing/Antioxidant Power (FRAP) Assay

The FRAP (ferric reducing/antioxidant power) assay according to Benzie et al. protocol (28). The data was expressed as millimoles of ferric ions reduced to the ferrous form per liter (29).

#### 3.5.5. Protein Assay

Total protein concentration was measured by Bradford’s method, using bovine serum albumin as the standard (4).

### 3.6. Statistical Analysis

Results were analyzed using SPSS version 15 and expressed as mean ± SEM. Comparisons among groups were performed using one way ANOVA and repeated measurement ANOVA followed by LSD for multiple comparison tests. P-values of less than 0.05 were considered significant.

## 4. Results

### 4.1. LDH, CK and CK-MB Activity

**Figure 1.**
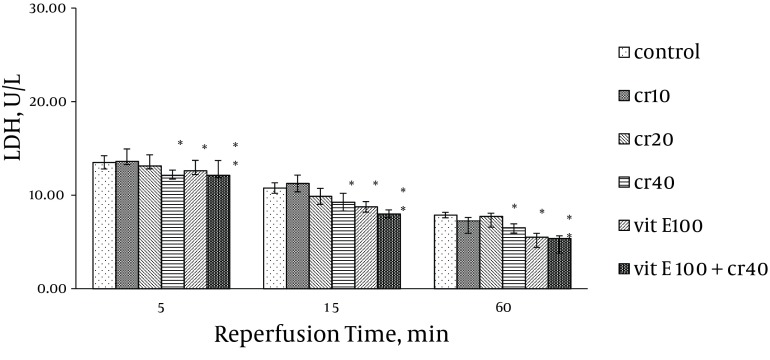
Effect of Crocin and Vitamin E on LDH Activity in Coronary Effluent Thirty minutes ischemia followed by 60 minutes reperfusion was induced in all groups. Data expressed as Mean ± SEM; n = 10; * P < 0.05; ** P < 0.01 were compared with the control group. Repeated measurement ANOVA was used, followed by LSD test.

**Figure 2.**
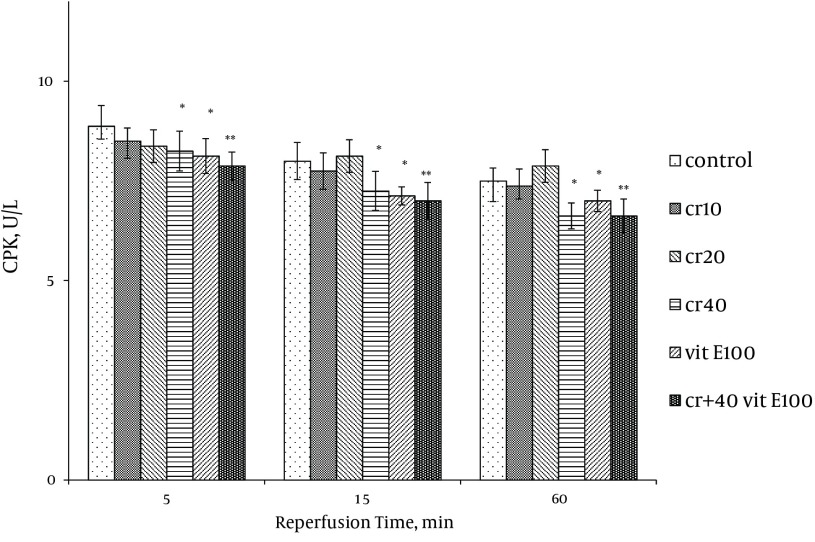
Effect of Crocin and Vitamin E on CPK Activity in Coronary Effluent After 30 Minutes of Ischemia Followed by 60 Minutes of Reperfusion Data expressed as Mean ± SEM; n = 10; * P < 0.05; ** P < 0.01 were compared with the control group. Repeated measurement ANOVA was used, followed by LSD test.

**Figure 3.**
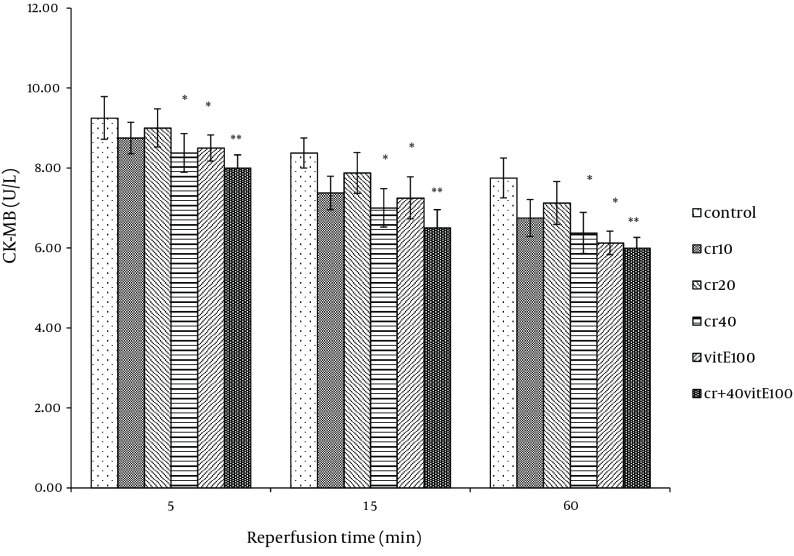
Effect of Crocin and Vitamin E on CK-MB Activity in Coronary Effluent After 30 Minutes of Ischemia Followed by 60 Minutes of Reperfusion Data expressed as Mean ± SEM; n = 10; * P < 0.05; ** P < 0.01 were compared with the control group. Repeated measurement ANOVA was used, followed by LSD test.

Cardiac biomarkers activity was measured in the coronary effluent as index of myocardial cellular injury after ischemia-reperfusion and showed elevation during reperfusion. In the rats treated with 40 mg/kg of crocin and 100 mg/kg of vitamin E, LDH activity was significantly lower at 15 and 60 minutes after reperfusion compared with the control (P < 0.05). The LDH activity with combination of crocin and vitamin E was more significantly lower than the control group (P < 0.01, Figure 1). Additionally, crocin significantly decreased the release of LDH from the myocardium. The CK (CPK) and CK-MB were also significantly (P < 0.01) (Figures 2, 3) reduced similar to LDH activity in the coronary effluent.

### 4.2. Thiobarbituric Acid Reactive Species Measurement

**Figure 4.**
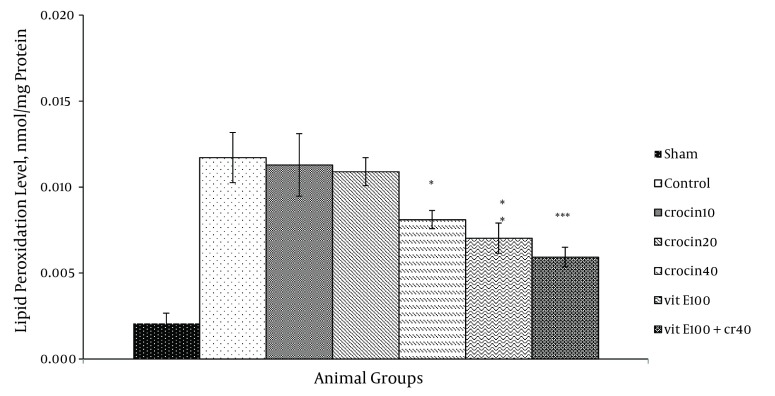
TBARS Levels in Rat Hearts After 30 Minutes of Ischemia and 60 Minutes of Reperfusion Data expressed as Mean ± SEM; n = 10; * P < 0.05; ** P < 0.01 were compared with the control group (normal saline). One-way ANOVA was used, followed by LSD test.

There was an increase in the MDA levels following ischemia-reperfusion. Pretreatment with 40 mg/kg crocin (P < 0.05) and 100 mg/kg vitamin E (P < 0.01) resulted in a significant reduction in the free radical-mediated lipid per oxidation as indicated by a decrease in the MDA levels (Figure 4). TBARS (thiobarbituric acid reactive species) levels were prominently reduced in combination treatment compared with the control group (0.006 ± 0.001 vs. 0.012 ± 0.001, P < 0.001).

### 4.3. Endogenous Antioxidant Enzymes

**Figure 5.**
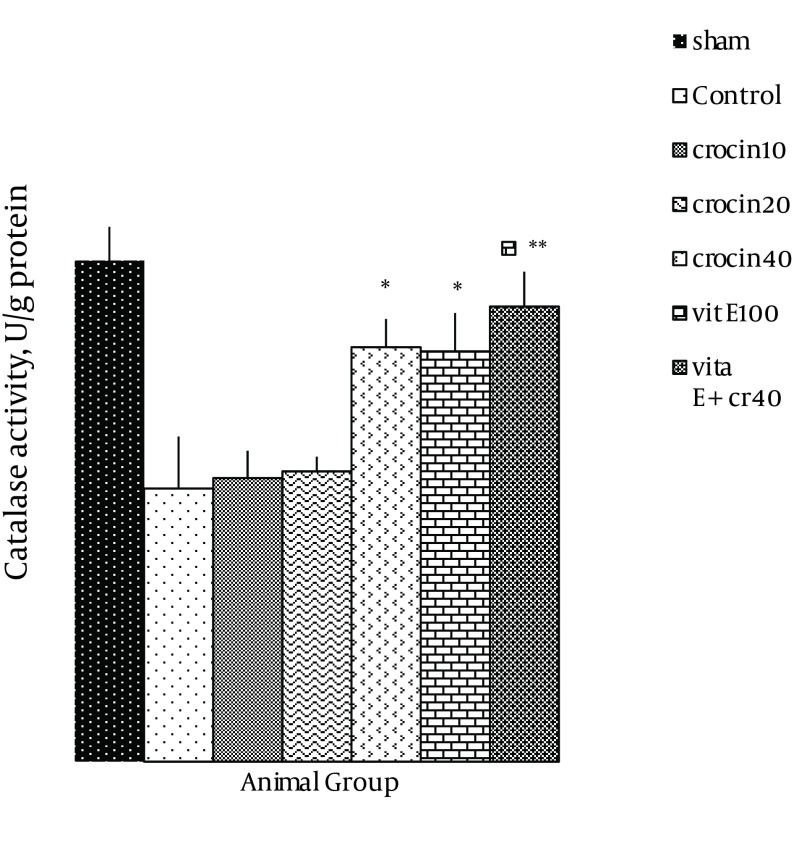
CAT Levels in Heart of Rats After 30 Minutes of Ischemia and 60 of Minutes Reperfusion Data expressed as Mean ± SEM; n = 10; * P < 0.05; ** P < 0.01 were compared with the control group. One-way ANOVA was used, followed by LSD test.

**Figure 6.**
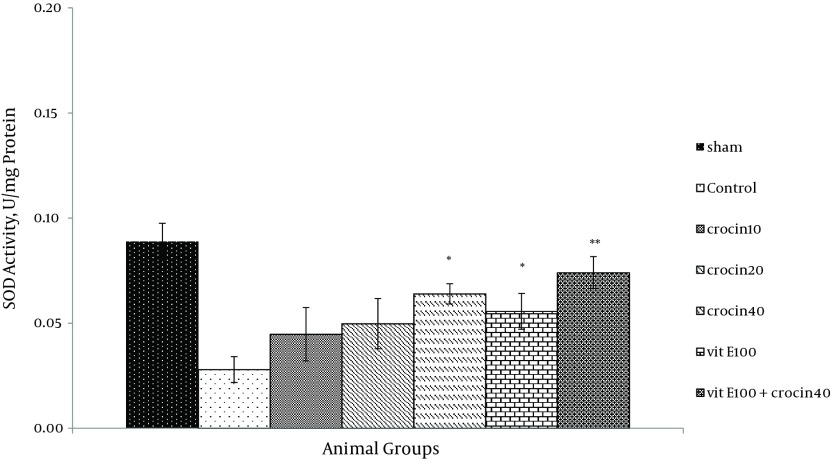
SOD Levels in Heart of Rats After 30 Minutes of Ischemia and 60 Minutes of Reperfusion Data expressed as Mean ± SEM; n = 10; * P < 0.05; ** P < 0.01 were compared with the control group (one-way ANOVA followed by LSD test).

There was no change in catalase activity in rat hearts exposed to ischemia-reperfusion compared with the sham group. The CAT level was higher than the control group in the rats, which received 40 mg/kg of crocin and 100 mg/kg of vitamin E (P < 0.05) (Figure 5) and a combination of crocin with vitamin E could partially restore the CAT activity to levels similar to that of the sham group (0.052 ± 0.004 vs. ctrl 0.031 ± 0.006, P < 0.01). Moreover, superoxide dismutase activity was reduced in the control group compared with the sham group (Figure 6). The SOD activity was also significantly reduced in rats which received crocin (40 mg/kg) compared with the control group (P < 0.05). Although vitamin E administration significantly reduced SOD activity compared with the control, a combination of crocin with vitamin E was more effective (0.074 ± 0.01 vs. 0.028 ± 0.006, P < 0.01).

### 4.4. Measurement of FRAP Value

**Figure 7.**
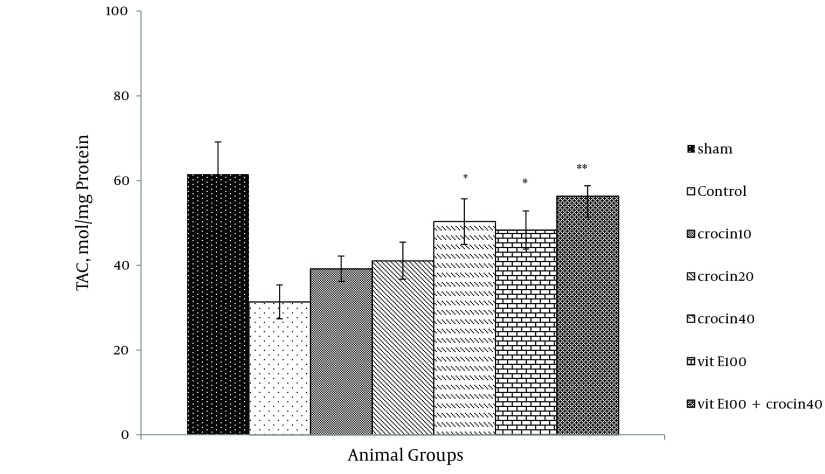
Total Antioxidant Capacity (TAC) Levels in Heart of Rats After 30 Minutes of Ischemia and 60 Minutes of Reperfusion Data expressed as Mean ± SEM; n = 10); * P < 0.05; ** P < 0.01 were compared with the control group (one-way ANOVA followed by LSD test).

Ischemia-reperfusion leads to a significant reduction in FRAP value of samples as compared with sham animals (31.4 ± 4.1 versus 61.49 ± 7.6 mmol/mg protein). Since the crocin and vitamin E pretreatment increased antioxidant power (Figure 7), a combination of crocin (40 mg/kg) with vitamin E was more significantly effective (56.3 ± 2.5 vs. 31.4 ± 4.1, P < 0.01).

## 5. Discussion

Scientists have made efforts to identify substances that can be used for management of clinical complications following cardiac ischemia-reperfusion injuries (15). The results obtained in the present study suggest that crocin as a carotenoid pigment has the same Protective effect in compare vitamin E against cardiac ischemia-reperfusion injury by altering antioxidant capacity in isolated rat heart model. Cardiac marker enzymes like CPK, CK-MB and LDH collected in coronary effluent were used as a predictor for pathological changes. These enzymes are released into the extracellular fluid during myocardial injury (30). The current results showed an increase in activity of these enzymes following ischemia-reperfusion. Moreover, it was observed that oral administration of crocin significantly decreased activities of CK-MB and LDH in heart tissue and thus, exhibited a protective effect on myocardium cell membrane integrity (21). A number of processes have been implicated with the pathogenesis of ischemia-reperfusion induced cell injury. These involve disturbances of cell calcium homeostasis, depletion of ATP, activation of enzymes like phospholipases, proteases and generation of free radicals that can cause oxidative damage to cellular macromolecules (31). The deleterious effect of these free radicals is mainly on the phospholipid membrane causing its degradation. Lipid peroxidation is an important pathogenic event in myocardial necrosis. In the present study, crocin significantly decreased MDA contents to almost normal levels. Several earlier reports have demonstrated a reduction in MDA levels by aqueous extracts of Saffron or its active constituents (crocin) in renal ischemia-reperfusion induced-injury in rats (15) and in ischemia-reperfusion model of stroke in rat brains (14). These results confirmed that crocin has a potential to reduce lipid peroxidation in the pathological condition ischemia-reperfusion induced oxidative stress. Under acute pathological conditions such as ischemia, the balance between oxidant and antioxidant systems changes (32). Therefore, FRAP assay was used to evaluate the antioxidant capacity of cardiac homogenate samples after ischemia-reperfusion. As expected, a significant reduction in antioxidant power, as indicated by the FRAP value, was observed. Instead, crocin increased the antioxidant power of heart homogenate samples (28). In this study, an increase in the levels of catalase and superoxide dismutase enzymes was observed after administration of crocin.

Some of the endogenous antioxidants including glutathione peroxidase, superoxide dismutase, and catalase act as a primary defense mechanism whereas the others such as vitamin E may play a secondary role in reducing ischemia-reperfusion injury. As a matter of fact, there are differences in the structures of crocin and vitamin E. Alpha-tocotrienol (natural vitamin E) has three double bonds in its lipophilic tail and crocin’s structure involves seven double bonds. Crocin as a carotenoid pigment of saffron is an antioxidant, which has the same potency as vitamin E against oxidative stress-induced ischemia-reperfusion in the rat isolated heart. These results suggest that crocin has an ability to suppress the generation of ROS by various oxidative stresses. However, the exact mechanism of action of crocin remains unknown. In conclusion, on the basis of the present biochemical studies, the optimal dose of crocin is 40 mg/kg. Crocin, as well as vitamin E, preserved cardiac contractile function by restoration antioxidant defense system, decreasing lipid peroxide formation and preserving activities of CK-MB and LDH enzymes. These results suggest that crocin, like vitamin E, not only decreases oxidative stress but may also delay the progression of ischemic heart disease.
